# The Role of Sphingomyelin and Ceramide in Motor Neuron Diseases

**DOI:** 10.3390/jpm12091418

**Published:** 2022-08-30

**Authors:** Gavin McCluskey, Colette Donaghy, Karen E. Morrison, John McConville, William Duddy, Stephanie Duguez

**Affiliations:** 1 Personalised Medicine Center, School of Medicine, Ulster University, Derry BT47 6SB, UK; 2 Department of Neurology, Altnagelvin Hospital, Derry, BT47 6SB, UK; 3 Department of Neurology, Royal Victoria Hospital, Belfast BT12 6BA, UK; 4 Faculty of Medicine, Health & Life Sciences, Queen’s University, Belfast BT9 6AG, UK; 5 Department of Neurology, Ulster Hospital, Dundonald, Belfast BT16 1RH, UK

**Keywords:** sphingomyelin, ceramide, Amyotrophic Lateral Sclerosis, sphingolipid, motor neuron disease

## Abstract

Amyotrophic Lateral Sclerosis (ALS), Spinal Bulbar Muscular Atrophy (SBMA), and Spinal Muscular Atrophy (SMA) are motor neuron diseases (MNDs) characterised by progressive motor neuron degeneration, weakness and muscular atrophy. Lipid dysregulation is well recognised in each of these conditions and occurs prior to neurodegeneration. Several lipid markers have been shown to predict prognosis in ALS. Sphingolipids are complex lipids enriched in the central nervous system and are integral to key cellular functions including membrane stability and signalling pathways, as well as being mediators of neuroinflammation and neurodegeneration. This review highlights the metabolism of sphingomyelin (SM), the most abundant sphingolipid, and of its metabolite ceramide, and its role in the pathophysiology of neurodegeneration, focusing on MNDs. We also review published lipidomic studies in MNDs. In the 13 studies of patients with ALS, 12 demonstrated upregulation of multiple SM species and 6 demonstrated upregulation of ceramides. SM species also correlated with markers of clinical progression in five of six studies. These data highlight the potential use of SM and ceramide as biomarkers in ALS. Finally, we review potential therapeutic strategies for targeting sphingolipid metabolism in neurodegeneration.

## 1. Introduction

Amyotrophic Lateral Sclerosis (ALS), Spinal Bulbar Muscular Atrophy (SBMA) and Spinal Muscular Atrophy (SMA) are progressive neurodegenerative conditions characterised by the progressive degeneration of motor neurons [1]. In ALS, both upper and lower motor neurons are affected, whereas SBMA and SMA are lower motor neuron (LMN) diseases [2]. Despite having different genetic and/or environmental causes, ages of onset and prognosis, these motor neuron diseases (MNDs) all exhibit progressive motor neuron dysfunction and cell death [1].

ALS, the most common adult-onset MND, has an incidence of approximately 1.6–3.8 per 100,000 [3]. Patients usually present aged 50–70 years, and the average survival from disease onset is 2–4 years [4]. Approximately 10% of cases are familial. Mutations in over 30 genes have been identified as causative or highly associated with ALS, most commonly in C9orf72, SOD1, FUS and TDP43 [4,5]. The genetic aetiology of ALS can now be identified for up to 67% of familial and 11% of sporadic cases [5].

SMA is caused by homozygous deletions or mutations in the Survival Motor Neuron 1 (SMN1) gene in 95% of cases. It affects an estimated 1 in 10,000 live births [6]. The SMN1 gene encodes the SMN protein, which is necessary for motor neuron survival. The SMN2 gene also encodes the SMN protein; however, a single nucleotide substitution results in the exclusion of exon 7 in almost 90% of SMN2 transcripts resulting in a fully functional SMN protein in only 10% of transcripts [7]. The clinical severity of SMA is largely determined by the copy number of SMN2 genes in patients, with those with a greater copy number having a milder clinical phenotype [8]. The majority (60%) of cases of SMA are SMA Type 1, the most severe type with disease onset at <6 months and a typical survival of 2 years [6,8]. However, the milder form of SMA, SMA Type 4 (<5% of cases), presents in adulthood and is associated with a normal life expectancy [8].

SBMA, also known as Kennedy’s Disease, is an X-linked recessive disease caused by a CAG trinucleotide repeat expansion in the Androgen Receptor (AR) gene [9]. Typically only males are affected, but female carriers may show mild manifestations such as cramps. It has a reported prevalence of 2.58 per 100,000 in males but is thought to be underdiagnosed [10]. It may be difficult to distinguish SBMA and ALS clinically, especially in cases of ALS with predominantly LMN features. Previous studies have shown that 2% of males who were clinically diagnosed with ALS actually had SBMA [11]. Patients usually present at age 30–60 and have a much slower progressive weakness than ALS [12]. In addition to the progressive motor deficit, patients with SBMA often have minor sensory neuropathies, which may be asymptomatic, and signs of androgen dysfunction such as gynaecomastia and infertility [10]. 

Lipid dysregulation has been described in each of these conditions [13]. While all lipid classes are reported to be dysregulated in ALS, sphingolipid (SL) metabolism has been described as the most dysregulated pathway, with the sphingomyelin-ceramide pathway a key regulator in neurodegeneration [14,15,16,17]. This review will briefly summarise how lipid metabolism is affected in MNDs. After a brief description of the metabolism and function of SLs, this review highlights their dysregulation in MNDs, focusing on ceramide and sphingomyelin. Finally, the potential use of ceramide and sphingomyelin as biomarkers for MNDs and the possibility of targeting SM pathways as a therapeutic strategy for MNDs is explored.

## 2. Lipid Dysregulation in MNDs

### 2.1. Dyslipidaemia in ALS

Hypermetabolism, defined as an excessive increase in energy expenditure, is well documented in ALS and is associated with shorter survival [18,19]. The cause is multifactorial, with a combination of multiple factors including increased energy expenditure, mitochondrial dysfunction and altered glucose/insulin and lipid metabolism, as well as hypothalamic dysfunction [20]. Dyslipidaemia with high LDL/HDL ratios, as well as high BMI and subcutaneous fat, is associated with a slower rate of functional decline (measured by the revised ALS Functional Rating Scale (ALSFRS-R)) and longer survival in ALS [21,22,23]. Higher HDL and apolipoprotein A1 levels reduce the risk of developing ALS [24]. Although beyond the scope of this review, there is also considerable literature, some of it conflicting, on the role of statins, known modulators of lipid metabolism, both in increasing the risk of ALS and in potential effects on increasing the progression of the disease. Some studies have shown an increased rate of functional decline and muscle cramps in patients taking statins [25]. Others have suggested an increased risk of ALS in patients taking statins [26]. However, other studies, including systematic reviews and meta-analyses, have not supported these findings [27,28,29]. 

### 2.2. Hyperlipidaemia in SBMA

Patients with SBMA also display lipid dysregulation with high rates of hyperlipidaemia, the metabolic syndrome, and of non-alcoholic fatty liver disease (NAFLD) [30,31]. ARs are known to modulate lipid metabolism by mediating the cellular effects of testosterone [32]. Longer CAG repeats inversely correlate with the transcriptional activity of testosterone target genes and positively correlate with BMI, body fat and fat-free mass [33,34]. Lipid dysregulation and altered expression of lipid-regulating genes have been identified prior to the onset of denervation in mice with SBMA [35].

### 2.3. Dysregulated Fatty Acid Metabolism in SMA

Patients with SMA have been shown to have dysregulated fatty acid metabolism, increased rates of NAFLD and increased free fatty acid levels [36,37,38]. SMA mouse models have been shown to have 25-fold increases in hepatic triglyceride levels compared to controls, along with a global dysregulation of fatty acid metabolism [38]. It has been suggested that denervated muscle in SMA exacerbates the increased circulating fatty acid levels due to its non-functional state and changes in the metabolism of atrophic muscle with reduced capacity for fatty acid oxidation [36].

## 3. Sphingolipid Synthesis

**Figure 1:**
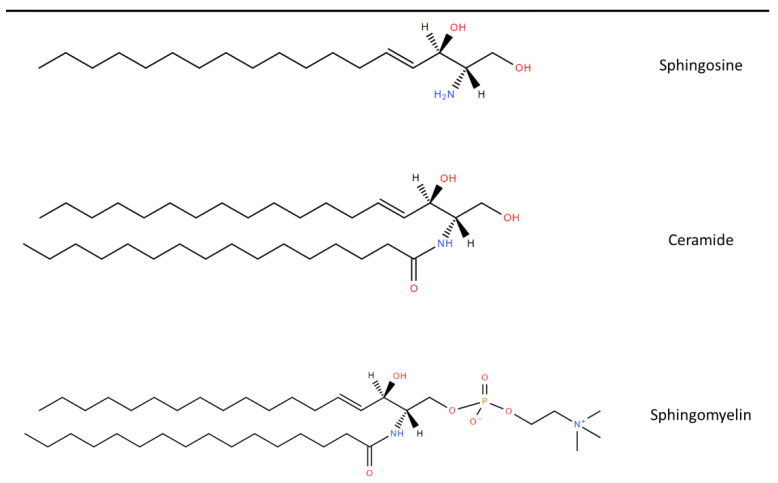
Chemical structure of common Sphingolipids. Sphingosine is the most common long-chain base. A fatty acid acyl chain is connected to the C2 amide group to form Ceramide and then Sphingomyelin is formed by the subsequent addition of a phosphocholine head group. Lipid structures created using LIPID MAPS^®^ tools [43].

Sphingolipids (SLs) are a diverse class of lipids with eighteen carbon amino-alcohol backbones, which are synthesized in the ER from non-sphingolipid precursors [39]. They play significant roles in membrane structure and have many bioactive metabolites, which regulate cellular function [39,40]. The basic structure of SLs is ceramide. Ceramide consists of a sphingoid long-chain base and a fatty acid acyl chain connected to an amine bond [41]. The most common mammalian long-chain base is sphingosine (d18:1), an 18 Carbon chain with a trans double bond at positions 4–5 [42]. The structure of sphingosine, ceramide and SM are shown in Figure 1.

### 3.1. Ceramide Metabolism

**Figure 2:**
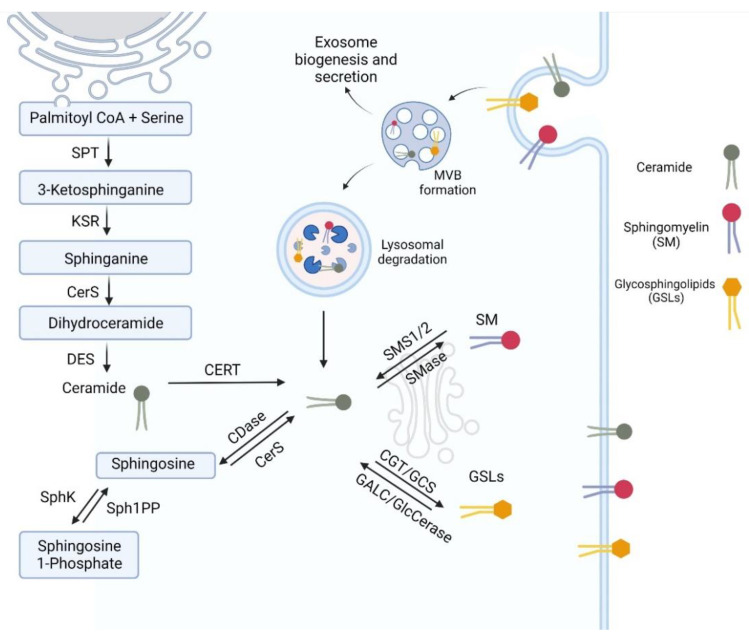
Sphingolipid (SL) metabolism showing de novo ceramide synthesis at the endoplasmic reticulum. It is then transferred to the Golgi apparatus where it can be modified to complex SLs and subsequently transported to the plasma membrane. There is recycling of SLs through the endosome, then multivesicular body (MVB) formation and finally lysosomal degradation. Ceramide can be further broken down into sphingosine and spingosine-1-phosphate. SPT—serine palmitoyl transferase, KSR—3-Ketosphinganine reductase, CerS—ceramide synthase, DES—dihydroceramide desaturase, CERT—ceramide transfer protein, CDase—ceramidase, SphK—spingosine kinase, Sph1PP—Sphingosine-1-Phosphate Phosphatase, SMS—sphingomyelin synthase, SMase—sphingomyelinase, GCT—ceramide galactosyltransferase, GCS—glucosylceramide synthase, GALC—galactosylceramidase, GlcCerase—glucosylceramidases, SM—sphingomyelin, GSL—glycosphingolipids. Figure created with BioRender.com.

Ceramide metabolism and the metabolism of complex SLs are summarized in Figure 2. Ceramide is synthesized de novo in the endoplasmic reticulum in a series of steps. Firstly, Serine and Palmitoyl CoA (a 16-chain fatty acid) are converted into 3-Ketosphinganine by serine palmitoyl transferase (SPT), and then 3-ketosphinganine is reduced to sphinganine by 3-Ketosphinganine reductase (KSR) [39,44]. At this stage, sphinganine is either acylated by ceramide synthase (CerS) to form dihydroceramide or phosphorylated by sphingosine kinase to form sphingosine 1-phosphate (S1P). There have been six different CerS identified, each with a preference for binding fatty acids with different acyl chain lengths to sphinganine, explaining the diversity of acyl chain length in SLs [41]. Dihydroceramide is then desaturated by dihydroceramide desaturase to form ceramide. Ceramide is then transported to the Golgi apparatus for further modifications into complex SLs [39]. Ceramide has low solubility in aqueous environments and is a membrane-bound molecule. Cells must therefore actively transport it between membranes, and this is performed either by vesicular transport or the ceramide transfer protein (CERT). CERT has a preference for ceramide species with acyl chains less than C22 and is less efficient at transferring longer chain ceramides. Ceramides transferred to the trans-Golgi apparatus by CERT are preferentially used for sphingomyelin (SM) synthesis, whereas ceramides transferred by vesicles are transferred to the cis-Golgi region and predominantly used for glycosphingolipid synthesis [39,45].

Ceramide can also be deacylated to sphingosine by ceramidases and then phosphorylated to sphingosine-1-phosphate (S1P) by sphingosine kinase. These are reversible reactions, and S1P can then be converted back to sphingosine by sphingosine-1-phosphate phosphatase and ceramide subsequently created by CerS as described above [46].

### 3.2. Metabolism of Complex Sphingolipids

The most abundant complex SL is SM. Sphingomyelin Synthase (SMS) converts ceramide to SM through the addition of a phosphocholine head donated by phosphatidylcholine resulting in the formation of SM and diacylglycerol. There are two sphingomyelin synthases (SMS1 and SMS2) in the Golgi apparatus, and SMS2 is also located in the plasma membrane [44]. SM is broken back down to ceramide by sphingomyelinase (SMase). There are three major categories of SMase classified according to their optimum pH (acid, alkaline and neutral SMases), with different cellular distributions [47]. Alkaline SMase is expressed exclusively in the intestines and liver for dietary digestion of SM [48]. Acid SMase is predominantly a lysosomal SMase but is also secreted into the extracellular space. Neutral SMases are found in the nucleus, ER, Golgi apparatus and plasma membrane [49]. In addition to their function in degrading SM to ceramide, neutral SMases are involved in the secretion of extracellular vesicles [50]. 

The other complex SL is glycosphingolipids (GSL). More than 400 different glycans have been identified linked to a ceramide backbone by a b-glycosidic bond, leading to a huge variety of structurally different GSLs [51]. Ceramide is converted to either galactosylceramide by ceramide galactosyltransferase (GCT) or to glucosylceramide by glucosylceramide synthase (GCS) [39]. These are the common precursors of all GSLs. Further modification leads to more complex GSLs, e.g., sulfatides, gangliosides, lactosylceramides and hexosylceramides [52]. Galactosylceramide and glucosylceramide can be converted back to ceramide by galactosylceramidase (GALC) and glucosylceramidases (GlcCerase), respectively [53]. More complex GSLs are degraded by lysosomal enzymes to recycle ceramide and are discussed in reviews of lysosomal storage disorders [54]. 

## 4. Biological Function of Ceramide and Sphingomyelin

**Figure 3:**
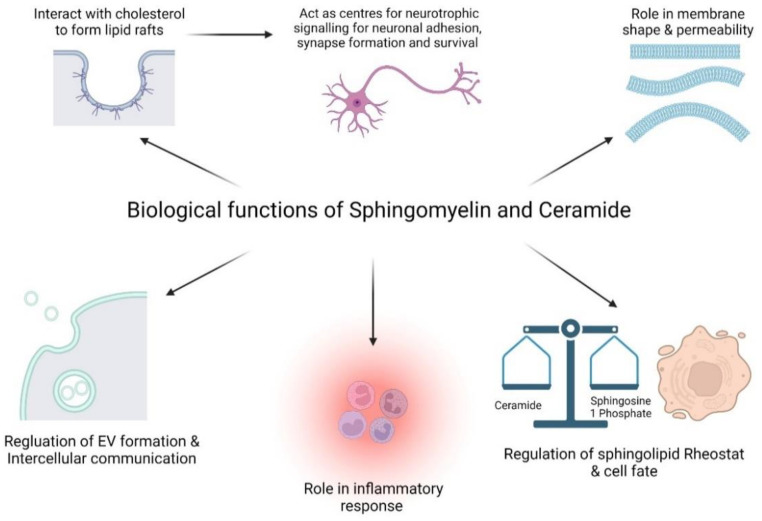
Sphingomyelin (SM) and ceramide functions. Figure created with BioRender.com.

SLs play important structural roles in cell membranes. SM is the most abundant SL in the plasma membrane. SMs vary in acyl chain length and saturation, and this affects the permeability, fluidity and structure of the plasma membrane [55]. SM interacts strongly with cholesterol. The main feature of the close sphingomyelin-cholesterol interaction is thought to be a hydrogen bond between the amide group of SM and the 3-hydroxyl group of cholesterol [56]. SM concentration in the plasma membrane affects cholesterol homeostasis [44]. Degradation of SM from the plasma membrane leads to cholesterol moving from the membrane to the ER where is it esterified, and also downregulates HMG-CoA reductase, a key rate-limiting enzyme in cholesterol synthesis [57]. SM interacts with cholesterol to form lipid rafts, which are lipid- and protein-rich domains in the extracellular leaflet of the membrane that exists in a liquid-ordered phase and serve to compartmentalize important cellular functions (Figure 3). These rafts exist in two forms: Calveolae, which are small invaginations (50–100 nm) in the membrane, and planar non-calveolar forms [55]. Lipid rafts in neurons form an organizing centre for neurotrophic signalling for processes that include neuronal adhesion, synapse formation and maintenance. They also contain the receptors for neurotrophins, a group of polypeptides, which activate signalling pathways for the development, function and survival of neurons [58]. Again, alterations in the SM chain length and saturation affect its ability to interact with cholesterol [59]. Shorter C16 SMs have higher solubility limits and form a greater number of liquid-ordered domains, which are larger and have greater thermostability than longer-chain C24 SMs, with a similar effect observed with unsaturated vs. saturated SMs [60]. Thus, the variation in the SM acyl chain is important for cellular processes. It is important to note the difficulties of analysing the structure and function of lipid rafts given their nanoscopic and dynamic nature [61]. Observations have been based on characteristics of plasma membrane models of different lipid mixtures and giant plasma membrane vesicles (GPMVs), which bud from plasma membranes [60,62]. GPMVs retain membrane lipid and protein diversity, and are capable of phase separation but only form optically resolvable lipid rafts at low temperatures of up to 20 °C [62]. More advanced techniques such as fluorescence resonance energy transfer have now allowed for the analysis of smaller rafts at physiological temperatures [63,64].

In addition to these roles, SM is also broken down to ceramide on the plasma membrane by neutral SMase (Figure 3). Ceramide also exists in the plasma membrane and in the lipid rafts discussed above. However, it also plays a more direct role in cell signalling. Ceramide and its metabolite S1P form a ‘sphingolipid rheostat’, which determines the cell fate [65]. Most ceramide species are thought to be pro-apoptotic and associated with cell death, whereas S1P promotes cell proliferation and survival. TNF alpha induces ceramide formation, a key step in TNF alpha-mediated apoptosis [66]. Ceramide induces apoptosis through the activation of the stress-activated protein kinase (SAPK) or inhibition of the mitogen-activated protein kinase (MAPK) pathways [67]. S1P inhibits ceramide-mediated apoptosis through the activation of the extracellular signal-regulated kinase (ERK) pathway, as well as counteracting the SAPK pathway [68]. Increased ceramide production has been seen in NB2a neuroblastoma cells in retinoic-acid-induced apoptosis [69]. Amyloid beta peptides have been shown to increase ceramide production through the induction of SMase and result in apoptosis in oligodendrocytes [70]. The same study found that preventing ceramide degradation through the inhibition of ceramidase also increased cell apoptosis. The production of ceramide by SMase has been shown to result in nerve growth factor (NGF)-mediated apoptosis in motor neurons overexpressing SOD1^G93A^ [17]. Blocking ceramide production from SM by inhibiting SMase was shown to prevent nerve-growth-factor-mediated cell death in hippocampal neurons [71]. Controlling the sphingolipid rheostat is therefore crucial in cell homeostasis. S1P also directs lymphocyte egress from lymph nodes, playing an important role in inflammation [72] (Figure 3).

SM and ceramide affect intercellular communication through the formation of extracellular vesicles (EVs) [73] (Figure 3). EVs are small vesicles enclosed in a lipid bilayer secreted from almost all cells and are detectible in a variety of biofluids [74,75]. They are involved in intercellular communication with both neighbouring and distant cells through the transfer of lipids, proteins and genetic material [76,77]. EVs are formed through two main pathways, the Endosomal Sorting Complex Required for Transport (ESCRT)-dependent and ESCRT-independent systems [73,78]. The ERSCT-independent pathway is a lipid-dependent process [79]. Plasma membranes have an asymmetric lipid distribution with SM and Phosphatidylcholine (PC) enriched on the luminal side [40]. The hydrolysis of SM to ceramide by SMases results in increased membrane fluidity and the cone-shaped structure of ceramide results in negative curvature of the membrane and subsequent Intraluminal Vesicles (ILV) formation [40,80]. S1P then activates receptors on Multivesicular Bodies (MVBs) to segregate ILVs for secretion as EVs [81]. Experimental support for this method of EV formation is that the stimulation and inhibition of neutral SMase2 increased and reduced EV secretion, respectively [82]. EV formation and biological functions are discussed in detail in other reviews [73].

## 5. Role of Sphingolipids in MNDs

Given the roles of SLs in many vital biological processes and their high abundance in the central nervous system as major components of oligodendrocytes and myelin sheaths, SL metabolism is thought to be a key pathway in neurodegeneration and neuroinflammation [83]. Alteration in SL metabolism has been linked to multiple neurodegenerative diseases such as Alzheimer’s Disease and Parkinson’s Disease, as well as neuroinflammatory conditions such as Multiple Sclerosis. These are discussed in detail in other reviews [84,85,86]. 

Increased levels of SM and ceramide have been found in spinal cord tissue of patients with ALS and SOD1 mice [16]. A study in the wobbler mouse, which is a model of motor neuron degeneration, identified the mis-sorting of lysosomal SL degradation enzymes with a resultant increase in SL intermediates [87]. Lipid dysregulation in ALS can occur decades before classical symptoms, and lipid biomarkers can be used to identify individuals at risk of developing ALS [24,88]. In keeping with this, increased levels of SLs were identified in spinal cords of ALS mice prior to the onset of clinical signs, and SM was demonstrated to mediate motor neuron death via oxidative stress [16]. A transcriptomic meta-analysis study on spinal cord tissue from SOD1 mice found that cholesterol, ceramides and eicosanoid pathways were altered early in the disease course [89]. This has also been shown in human studies, with SL alteration identified in plasma samples of patients who subsequently developed ALS [90]. Importantly, these studies suggest that alterations in SL occur before motor neuron degeneration and are therefore an upstream process in ALS pathophysiology.

Over 20 risk genes in ALS are involved in lipid raft homeostasis and ceramide metabolic pathways [58]. Mutations or abnormal DNA methylation have been found in genes encoding for enzymes necessary for SL synthesis in patients with ALS and SMA, as well as bovine SMA. These are shown in Table 1. In addition, mutations in ASAH1, which result in dysfunctional acid ceramidase, cause a non-5q form of SMA associated with progressive myoclonic epilepsy [91]. Mutations in the SPTLC1 gene are associated with juvenile ALS and Hereditary Sensory and Autonomic Neuropathy type 1 (HSAN1) [92,93]. This gene encodes for a subunit of SPT, the enzyme required for the first step of SL synthesis. C-terminal SPTLC1 variants cause the formation of atypical deoxysphingolipids and result in HSAN1 [94]. The ALS-causing variants map to a transmembrane domain, which interacts with negative regulators of SPT activity and results in unregulated SPT and excess SL synthesis [95]. Epigenomic studies have also shown abnormal DNA methylation in SGMS2, which encodes for SMS2, the enzyme for converting ceramide to SM [96]. The CAV1 gene, which encodes for calveolin 1, has also recently been identified as a risk modifying gene in ALS. Calveolin 1 is found in lipid rafts and ALS variants in CAV1 were shown to disrupt lipid raft formation in patient-derived lymphoblastoid cells [97].

Another mechanism of how SLs can affect MNDs is through intercellular communication. Neutral SMase2 affects EV secretion. This has been demonstrated by studies showing that stimulation of SMase2 with TNF alpha increases EV secretion and inhibiting it with 1 PDDC reduces EV secretion [82,98]. EVs are being increasingly investigated in ALS as mediators of intercellular transfer of neurotoxic proteins such as TDP 43, FUS and SOD1 [99,100]. EVs secreted by muscle cells from ALS patients have been shown to be toxic to motor neurons [101].

Further insight into the importance of SL metabolism in neurodegenerative diseases is evident from lysosomal storage disorders. These are a group of over 40 conditions with a combined prevalence of 1 in 7000–8000 live births [102]. These diseases all are the result of impaired lysosomal degradation of various metabolites and the consequent effects on cellular function [103]. Several involve the degradation of SLs and are termed sphingolipidoses. These are a group of autosomal recessive or X-linked conditions with defects in enzymes required for the catabolism of SLs [104]. The cellular impact of the conditions depends on the concentration of the relevant SL and the degree of enzymatic deficiency. The sphingolipidoses and their enzymatic defects and effects on SLs are shown in Table 1. They each have a broad and unique clinical phenotype. However, given that SLs are enriched in the nervous system, these conditions often have the predominant feature of severe progressive neurodegeneration [104,105].

## 6. Lipidomic Studies in MNDs

The lipid profiles in MNDs have been mainly assessed via metabolomic analysis. Table 2 lists all of the published metabolomic studies that have included lipidomic analysis to date, detailing a range of different SM and ceramides identified. This may in part be explained by the different samples studied and the differing mass spectrometry methodologies for quantifying metabolites. Two studies were performed using spinal cord tissue, nine using plasma, two using serum, and two using CSF samples. Of the 12 studies comparing ALS to controls, all identified changes in SM concentrations, with SM species being increased in 11 studies and decreased in the other. Six studies identified increases in ceramide species, with decreases in some ceramides reported in one of these. A study of only of ALS patients found that multiple SMs were able to predict markers of disease progression such as the ALSFRS-R, manual muscle testing and respiratory function [116]. Another metabolomic study in 28 patients with ALS and 30 controls reported that out of 317 metabolites, 50 were increased and 70 decreased in ALS, although the individual metabolites were not listed [117]. 

One study identified four lipids, including SM C18:2, which were elevated several years before symptom onset [90]. This is of particular relevance with the progress in developing genotype-specific treatments, such as antisense oligonucleotides (ASOs) for patients with SOD1 and C9orf72 mutations and the need for biomarkers to guide the optimal timing for commencing treatment [118,119,120]. The ATLAS trial is currently evaluating Tofersen, an ASO for SOD1, in presymptomatic patients who develop raised neurofilament light chain levels, a marker of neuronal damage that becomes elevated 6–12 months prior to symptoms [121]. Given that lipids including SM and ceramide are altered early in the disease course [16,88], they could be of use in identifying presymptomatic patients for potential treatments. In addition, Blasco et al. have shown how SL biomarkers could be incorporated into pharmaco-metabolomic studies [116]. Baseline and follow-up SL profiles could be used to (1) further validate their use as prognostic markers compared to common clinical measurements of disease progression (such as lung function and ALSFRS-R) and (2) determine if treatments lead to alterations in metabolite levels. 

There are little data on the lipidomic profile of other MNDs. There have been no lipidomic studies in SBMA. SBMA and SMA patients were included as neurological mimics in one study but were combined as part of a group containing other conditions such as cervical myelopathy and multiple sclerosis [122]. In a metabolomic study of patients with SMA, H-nuclear magnetic resonance-based metabolic profiling demonstrated diagnostic and prognostic utility, but individual metabolites were not listed [123]. Another metabolomic study in 108 patients with SMA showed 200 metabolites correlating with the modified Hammersmith functional motor scale, including 12 lipids. Only 1 lipid (SM (C24:1)) was among the top 20 metabolites identified [124]. 

## 7. Potential Therapeutics Targeting Sphingolipid Metabolism

The increasing knowledge of the importance of SLs in neurodegeneration has led to multiple animal and patient studies targeting SL metabolism, and this may lead to future treatments for MNDs [134]. Fingolimod phosphate, an S1P receptor modulator, is an established treatment for Multiple Sclerosis [135]. It is considered to primarily exert its therapeutic function by preventing the egress of lymphocytes from lymph nodes, thereby reducing the recirculation of autoreactive T-lymphocytes into the CNS [136]. However, it also crosses the blood–brain barrier and has been shown to have wider signalling effects in the CNS, including protecting neurons from excitotoxic death in vitro [137]. It has been demonstrated to improve the neurological phenotype and survival in SOD1 mice and has now proceeded to a phase 2a trial in ALS where it has shown safety and tolerability [138]. This is the only SL modulator that has been used in a clinical trial in ALS to date. 

Other future potential therapeutic options are drugs that modulate enzymes involved in SL metabolism. Inhibition of neutral SMase2 to reduce EV release has been attempted in murine models of Parkinson’s Disease, in which alpha synuclein spread was reduced and motor scores were improved, as well as in murine models of Alzheimer’s Disease, resulting in improved cognition [98,139]. Ambroxol hydrochloride, a glucocerebrosidase 2 inhibitor, has demonstrated delayed disease onset and improved survival in SOD1 mice [140]. Inhibiting SL synthesis with myriocin, an inhibitor of SPT, improved the neurological phenotype of wobbler mice, a model of motor neuron degeneration [87]. Myriocin has also been shown to restore muscle function and reduce inflammation in murine models of Duchenne Muscular Dystrophy [141].

Finally, gene editing therapies may be a potential future therapeutic option. A study on patient-derived fibroblasts expressing ALS-linked SPTLC1 variants has shown that small interfering RNAs can target excess SL production in vitro [95].

## 8. Conclusions

SLs, particularly SM and ceramide, play vital roles in the nervous system and are dysregulated in neurodegenerative diseases. Lipid dysregulation is a well-known feature of MNDs, and this review highlights abnormalities in SM and ceramide, particularly in ALS. Multiple metabolomic studies have found that SM and ceramide species show utility as diagnostic biomarkers in ALS, and several also correlate with clinical measures of disease progression. Advancing knowledge of the role of SLs in neurodegeneration is leading to the investigation in animal models of drugs targeting SL metabolism, some of which are now progressing to clinical trials with the hope of translation into future therapies for patients. Future clinical trials could incorporate the evaluation of SLs to further validate their use as predictors of disease progression and to determine any effects of therapeutics on SL metabolism.

## Figures and Tables

**Table 1:** Abnormalities of Sphingolipid metabolism in Motor Neuron Diseases and sphingolipidoses.

Condition	Gene	Affected Enzyme/Protein	Effect on Sphingolipids
Sphingolipid synthesis			
Juvenile ALS [92] HSAN1 [93]	SPTLC1	SPT	Atypical deoxysphingolipids, cannot be converted into complex SLs or degraded
Bovine SMA [106]	FVT1	KSR	Reduced ceramide synthesis from de novo pathway
ALS type 8 [107] Late onset SMA [108]	VAPB	VAPB with effect on CERT and FAPP2	Impaired transfer of ceramide and glucosylceramide from ER to golgi apparatus
ALS [96]	SGMS2	SMS2	Affects sphingomyelin synthesis
Sphingolipid degradation			
SMA-PME [91] Farber’s disease [109]	ASAH1	Acid ceramidase	Ceramide accumulation
GM1 gangliodosis [110]	GLB1	β-Galactosidase	GM1 ganglioside accumulation
GM2 gangliodoses [110] Tay Sachs Disease Sandhoff’s Disease	HEXA HEXB	Hexaminidase A Hexaminidase A & B	GM2 ganglioside accumulation GM2 ganglioside, glycolipid GA2 and globoside accumulation
Fabry’s Disease [111]	GLA	α-Galactosidase A	Globotriaosylceramide accumulation
Metachromatic Leukodystrophy [112]	ARSA	Arylsulphatase A	Sulfatides accumulation
Niemann-Pick Disease [113] Type A & B Type C	SMPD1 NPC1/NPC2	Sphingomyelinase	Sphingomyelin accumulation
Gaucher’s Disease [114]	GBA	Glucocerebrosidase	Glucosylceramide accumulation
Krabbe’s Disease [115]	GALC	Galactosylceramidase	Galactosylceramide accumulation

HSAN1—hereditary sensory and autonomic neuropathy type 1, SPT—Serine palmitoyltransferase, VAPB—Vesicle associated membrane protein B, CERT—ceramide transfer protein, FAPP2—four phosphate adapter protein 2, SMS2—sphingomyelin synthase 2, SMA-PME—spinal muscular atrophy and progressive myoclonic epilepsy.

**Table 2:** Metabolomic studies in patients with ALS showing the changes in lipid metabolites.

Study	Patients	Sample Type	Quantification Platform	Metabolites Evaluated	Lipid Changes in MND	Prognostic Use
Blasco et al. 2017 [125]	40 ALS 45 Controls	CSF	HRMS	122 lipids	↑: PC (36:4p), PC (36:4e), SM (d43:2), SM (d34:0)	Higher SM (d43:2) and lower TG (16:0/16:0/18:1) and TG (18:0/16:0/18:1) had slower progression
					↓: TG (16:1/18:1/18:2)	
Lawton et al. 2012 [126]	161 ALS 117 Controls	Plasma	GC/MS and UPLC-MS/MS	335 lipids, proteins and carbohydrates	↑: LPC (16:1) and SM (18:0)	Not evaluated
Cutler et al. 2002 [16]	9 ALS 3 Control	Spinal cord	ES/MS/MS	Sphingolipids, Phospholipids, Cholesterol Esters, and Lipid Peroxides	↑: Cer (C16:0), Cer (C24:0), SM (C16:0), CE (C16:0) and CE (C18:0)	Not evaluated
Goutman et al. 2020 [127]	125 ALS 71 Controls	Plasma	UPLC-MS/MS	899 metabolites	↑: 8 Cers, 28 DAGs, 5 HEXC, 24 SMs,	Not evaluated
					↓: 5 DAGs, 5 SMs	
Goutman et al. 2022 [128]	Above cohort of 125 ALS and 71 controls with 2nd cohort 225 ALS, 104 controls	Plasma	UPLC-MS/MS	640 metabolites	SM most significant sub-pathway LCFA, acyl intermediates and Cers also raised	SM (d18:1/24:0), SM (d18:1/20:0, d16:1/22:0), SM (d18:1/14:0, d16:1/16:0) and lignoceroylcarnitine (C24) correlated with ALSFRS-R
Bjornevik et al. 2019 [90]	275 ALS 549 Controls	Plasma	LC/MS	404 metabolites	↑: SM (C18:2), PC (C40:7), PC (C38:4), CE (C22:4)	Not evaluated
					↓: 12 TAGs, DAG (C36:1), DAG (C36:2), PC (C36:2), 21-deoxycortisol, butyrobetaine	
Lawton et al. 2014 [122]	172 ALS 73 neurological mimics 50 Controls	plasma	GC/MS and UPLC-MS/MS	367 metabolites	↑: SM (d18:1/16:0), 5 FAs, 3-dehydrocarnitine, 1,2-propanediol, Chol, 1-stearoyl-GPI	1,2-propanediol correlated with ALSFRS-R
Chang et al. 2021 [129]	36 ALS 36 Controls	plasma	LC–MS/MS	185 metabolites	↑: SM (C24:1), SM (C20:2), PC (C44:5), PC (C34:2)	14 PCs and (OH) SM(C22:1) correlated with ALSFRS-R
					↓: (OH) SM(C22:1) (OH) SM(C24:1) 29 other PCs	
Fernandez-Eulate et al. 2020 [130]	20 ALS 20 Controls	Serum	UPLC-MS	416 lipids	↑: SM (39:1), SM (33:1), PE (P-20:1/0:0), PE (O-16:0/0:0), 5 PCs, androsterone, etiocholanolone and 2 FAs	Not evaluated
Blasco et al. 2018 [116]	74 ALS	Plasma	HPLC-MS/MS	188 metabolites	Not evaluated—no control participants	SM (C22:3) and SM (C34:1) correlated with disease progression, SM (24:1), SM (C16:1) and (OH) SM (C22:2) correlated with SVC
Dodge et al. 2015 [131]	6 ALS 6 Control	Spinal cord	LC-MS/MS	Cer, SM and GSLs	↑: Cer (C18:0), Cer (C24:1), (OH) Cer (C24:0), Cerebroside (C18:0 and C24:1), GlcCer (C18:0 and C24:1), LacCer (18:0), GL3 (C22:1), GM3 (C23:0), GM1 (C18:0) AND SM (C18:0)	Not evaluated
Sol et al. 2021 [132]	23 ALS 10 Controls	CSF Plasma	LC-MS/MS	1018 lipids in plasma and 843 in CSF	↑: 3 Fas, 2 DAGs, 13 TGs, 17 GPLs, 3 Cer, 1 SM	Fast vs. slow progressors had increased- 1 FA, 4 GLs, 4 GPLs, 2 Cer, 1 GM3, and decreased- 46 GLs, 36 GPLs, 2 Cer, 8 SM, 5 CE
					↓: 2 DAGs, 4 GPLs, 3 Cer, 3 GLs	
Area-Gomez et al. 2021 [133]	40 ALS 28 PLS 28 Control	Serum/Plasma	LC/MS	532 lipids	↑: Cer, LacCer, CE	SM declined and Cer increased at follow up
					↓: SM, PC, PS	

HRMS—high-resolution mass spectrometry, GC/MS—gas chromatography/mass spectrometry, LC/MS—liquid chromatography/mass spectrometry, LC–MS/MS—liquid chromatography/tandem mass spectrometry, UPLC-MS/MS—ultra-high-performance liquid chromatography/tandem mass spectrometry, ES/MS/MS—electrospray ionization tandem mass spectrometry, CSF—cerebrospinal fluid, ALSFRS-R—Revised ALS Functional Rating Scale, SVC—slow vital capacity, SM—sphingomyelin, TG—triglyceride, LPC—palmitoleoyl-glycerophosphocholine, Cer—ceramide, CE—cholesterol ester, DAG-Diacylglycerol, HEXC—hexosylceramide, LCFA—long chain fatty acid, TAG—Triacylglycerol, PC—phosphatidylcholine, FA—fatty acids, GPI—glycophosphatidylinositol, (OH)SM—hydroxysphingomyelin, PE—phosphatidylethanolamines, PS—phosphatidylserines, GPL—glycerophospholipids, GL—glycerolipid.

## Data Availability

Not applicable.
